# Distribution and Respiratory Activity of Mycobacteria in Household Water System of Healthy Volunteers in Japan

**DOI:** 10.1371/journal.pone.0110554

**Published:** 2014-10-28

**Authors:** Tomoaki Ichijo, Yoko Izumi, Sayuri Nakamoto, Nobuyasu Yamaguchi, Masao Nasu

**Affiliations:** 1 Environmental Science and Microbiology, Graduate School of Pharmaceutical Sciences, Osaka University, Suita, Osaka, Japan; 2 Research Institute for Humanity and Nature, Kyoto, Japan; Cambridge University, United Kingdom

## Abstract

The primary infectious source of nontuberculous mycobacteria (NTM), which are known as opportunistic pathogens, appears to be environmental exposure, and it is important to reduce the frequency of exposure from environmental sources for preventing NTM infections. In order to achieve this, the distribution and respiratory activity of NTM in the environments must be clarified. In this study, we determined the abundance of mycobacteria and respiratory active mycobacteria in the household water system of healthy volunteers using quantitative PCR and a fluorescent staining method, because household water has been considered as one of the possible infectious sources. We chose healthy volunteer households in order to lessen the effect of possible residential contamination from an infected patient. We evaluated whether each sampling site (bathroom drain, kitchen drain, bath heater pipe and showerhead) have the potential to be the sources of NTM infections. Our results indicated that drains in the bathroom and kitchen sink are the niche for *Mycobacterium* spp. and *M. avium* cells were only detected in the bathtub inlet. Both physicochemical and biologic selective pressures may affect the preferred habitat of *Mycobacterium* spp. Regional differences also appear to exist as demonstrated by the presence (US) or absence (Japan) of *Mycobacterium* spp. on showerheads. Understanding of the country specific human activities and water usage will help to elucidate the infectious source and route of nontuberculous mycobacterial disease.

## Introduction

Nontuberculous mycobacteria (NTM) species can be opportunistic pathogens, causing pulmonary disease, skin lesions, cervical lymphadenitis, and other health issues. In Japan, reported cases of NTM infection numbered 5.7 per 100,000 persons in 2005 [1]. An increase in the prevalence of NTM infection has been reported [2], [3], and in many countries, exceeds that of *Mycobacterium tuberculosis* [4]. Accurate incidence data, however, is not available, because NTM infections are not communicable between people and thus not reportable in industrialized countries such as the United States and Japan [1], [2]. Therefore, it is likely that more people than estimated may be infected with NTM. The source and route of NTM infections have been documented [5], [6], [7] but further research is needed to expand our understanding of this field.

NTM are present in various environments including freshwater, soil, household plumbing, and drinking water distribution systems [8]. The primary infectious source of NTM appears to be environmental exposure and there is no evidence for significant person-to-person transmission. As an alternative to antibiotic therapy, one alternative approach would be to reduce the frequency of exposure from environmental sources for preventing NTM infections. In order to achieve this, the distribution, abundance and activity of NTM in the environments must be clarified.

Culture methods are widely used for the detection and isolation of NTM in the environments [9]–[11]. However, there are some limitations in applying to environmental samples (i.e. hard to culture, overgrowth of nontarget bacteria [12], slow growth, variation in growth rate). Recently, quantitative PCR (qPCR) has been used to determine the abundance of NTM in aquatic environments [13]–[16]. qPCR, by its nature, is quantitative and rapid, but it does not detect physiological activity of mycobacteria. Previously, we therefore have developed a new method for rapid enumeration of respiratory active mycobacterial cells by acid-fast bacilli staining (Auramine O) following fluorescent vital staining (CTC [5-cyano-2, 3-ditolyl tetrazolium chloride] staining) [17]. CTC is used as an indicator of respiratory activity [18].

In this study, we determined the abundance of mycobacteria and respiratory active mycobacteria in the household water system of healthy volunteers using quantitative PCR and the previously developed fluorescent vital staining method (Auramine O-CTC double staining method). We also determined the abundance of *Mycobacterium avium*, in addition to the genus level. In many countries, *Mycobacterium avium* and *M. intracellulare* are the major species of clinically isolated NTM [19], [20]. Household water has been considered as one of the possible infectious sources, because of the similarity in genotypes between environmental isolates from patients' residential settings and clinical isolates (i.e. shower water and showerhead biofilm [5], bathroom and hot bath tub [6], [7]). They, however, focused on mycobacterial cells isolated from patients' residences, and it is difficult to distinguish mycobacterial cells in patients' residences from the cells which were originated from patients. We therefore chose healthy volunteer households for study in order to lessen the effect of possible residential contamination from an infected patient. We evaluated whether each sampling site (bathroom drain, kitchen drain, bath heater pipe and showerhead) have the potential to be the infectious sources of NTM infections.

## Materials & Methods

### Sampling sites in residential environments

Biofilm samples were collected from 5 sites in each of 40 residences using a sterilized polyester swab (Large Alpha Swab TX714A; Texwipe, Kernersville, NC). The 5 sites were bathroom drain, kitchen drain, bathtub inlet and outer and inner surfaces of showerhead, where biofilm are easily formed, and collected 38, 39, 27, 39 and 23 samples from each site, respectively (Table S1, Table S2). Sampling from each site was conducted under the agreement of volunteers. Each sample was suspended in 10 ml sterile water. In the traditional Japanese bath, a bathtub inlet is installed inside the bathtub and connects to a hot water supply or a boiler. However, the number of houses which is installed a western-style bathtub (no bathtub inlet) is increasing, the number of sample of bathtub inlet were less than those of others.

### Auramine O-CTC staining and enumeration of respiratory active mycobacterial cells

10 µl of bacterial suspension were spotted onto a microscopic glass slide (Matsunami Glass, Osaka, Japan) and mixed with an equal volume of 32 mM CTC (Dojindo Laboratories, Kumamoto, Japan). After 30 min-staining and drying with vacuum, the cells were stained with Rapid Modified Auramine O Stain Set (Scientific Device Laboratory, Des Plaines, IL) for 5 min and decolorized for 1 min. Finally, fluorescent images were captured by a cooled charged device camera (Retiga-2000R; Qimaging, Surrey, BC, Canada) mounted on an epifluorescence microscope (E-400; Nikon, Tokyo, Japan) with the Nikon filter sets B2-A (EX450-490, DM505, BA520), and respiratory active mycobacterial cells were enumerated by image analysis software BACS II [21].

### Direct DNA extraction

The bacterial suspension was filtered onto a 0.45 µm-pore size sterilized polycarbonate membrane filter (Advantec, Tokyo, Japan). DNA was extracted and purified by the methods reported by Tsai and Olson [22].

### Quantitative PCR for determination of bacterial abundance

For determination of abundance of total bacteria, *Mycobacterium* spp. and *M. avium*, the 16S rRNA gene was quantified with specific primers and probes using a LightCycler (Roche Diagnostics, Mannheim, Germany) (Table 1).

**Table 1:** Primers and probes used for quantitative PCR.

Target	Name	Primer or probe	Sequence (5'-3')	Reference
Bacteria	EUB f933	Primer	GCACAAGCGGTGGAGCATGTGG	23
	EUB r1387	Primer	GCCCGGGAACGTATTCACCG	23
*Mycobacterium* spp.	LC5	Primer	GGCGGAGCATGTGGATTA	24
	LC4	Primer	TGCACACAGGCCACAAGGGA	24
	LC39^a^	Probe	GCAACGCGAAGAACCTTACCTGG	24
	LC40^b^	Probe	TTTGACATGCACAGGACG	24
*M. avium*	*M. avium* FWD-01	Primer	CCTCAAGACGCATGTCTTC	25
	MAC REV-02	Primer	ACCTACCGTCAATCCGAGAA	25

a LC39 was labeled with fluorescein at the 3' terminus.

b LC40 was labeled with LCRed640 at the 5' terminus.

Total bacterial cells and mycobacterial cells were enumerated using the protocol reported by Yamaguchi *et al*. [23] and Lachnik *et al*. [24], respectively. For quantification of *M. avium*, we used the 16S rRNA gene targeted primersets [25]. To determine the DNA recovery rate during extraction, a known amount of PCR products of the luciferase gene (*luc*) were inoculated into the samples as an internal standard and quantified after DNA extraction as described previously [26]. The copy number of the 16S rRNA gene in a sample was calibrated based on the DNA recovery rate.

## Results

### Abundance of mycobacterial cells in household water system

The abundance of *Mycobacterium* spp. and *M. avium* is shown in Table 2. The Ribosomal RNA Operon Copy Number Database (rrndb) [27] showed that the average copy number of 16S rRNA gene possessed by Bacteria, *Mycobacterium* spp., *M. avium* was 4.2, 1.3 and 1, respectively (As of 8 Oct., 2013). Bacterial abundance (unit: cells/cm^2^) can be estimated from the results obtained with quantitative PCR (unit: copies/cm^2^) by using average copy number. The quantification limit in this study was determined as 10^1^ cells/cm^2^ from the standard curves for quantitative PCR. *Mycobacterium* spp. were frequently detected in bathroom drains (29 residences: 76%), bath tub inlets (12 residences: 44%) and kitchen drains (19 residences: 49%) (Table 2). On the other hand, detection frequency of mycobacterial cells in showerheads is much lower than those in other sampling site (outer surface, 1 residence: 3%; inner surface, 3 residences: 13%) (Table 2). The relative abundance of genus *Mycobacterium* to total bacteria in biofilm is shown in Table 3. 10 bathroom drain samples, three bathtub inlet samples, three kitchen drain samples and two inner showerhead samples showed that genus *Mycobacterium* was dominate (>10% of total bacteria). *M. avium* was only detected from bathtub inlets in five residences (19%) at the range of 10^1^–10^4^ cells/cm^2^ (Table S1).

**Table 2:** Number of residences where *Mycobacterium* spp. were detected in household water system.

Abundance a	Bathroom drain	Bathtub inlet	Kitchen drain	Showerhead (outer)	Showerhead (inner)
>10^7^ cells/cm^2^	0	0	1	0	0
10^6^–10^7^ cells/cm^2^	5	1	1	0	0
10^5^–10^6^ cells/cm^2^	7	1	12	0	0
10^4^–10^5^ cells/cm^2^	10	5	3	0	2
10^3^–10^4^ cells/cm^2^	4	4	1	0	0
10^2^–10^3^ cells/cm^2^	2	1	1	0	1
10^1^–10^2^ cells/cm^2^	1	0	0	1	0
<10^1^ cells/cm^2^	9	15	20	38	20

a Limit of quantification: 10^1^ cells/cm^2^.

**Table 3:** Relative abundance of *Mycobacterium* spp. as percentage of total bacteria in household water system.

Proportion	Bathroom drain	Bathtub inlet	Kitchen drain	Showerhead (outer)	Showerhead (inner)
>50%	3	1	1	0	2
40–50%	2	1	0	0	0
30–40%	0	0	0	0	0
20–30%	2	0	0	0	0
10–20%	3	1	2	0	0
1–10%	12	5	7	0	0
<1%	14	13	28	2	4
Not determined a	2	6	1	37	17

a Number of total bacteria was below quantification limit (10^1^ cells/cm^2^).

### Respiratory active mycobacteria in household water system

Figure S1 shows the microscopic image of a bathroom drain sample stained with Auramine O and CTC. Image analysis was used to determine the abundance of respiratory active mycobacteria (Table 4). In both types of drain samples, respiratory active mycobacterial cells were detected frequently (21% from bathroom drains and 12% from kitchen drains), and the abundances were relatively higher than those in bathtub inlet and showerhead.

**Table 4:** Number of residences where respiratory active *Mycobacterium* spp. were detected in household water system.

Abundance a	Bathroom drain	Bathtub inlet	Kitchen drain	Showerhead (outer)	Showerhead (inner)
>10^6^ cells/cm^2^	0	0	1	0	0
10^5^–10^6^ cells/cm^2^	3	0	0	0	0
10^4^–10^5^ cells/cm^2^	0	3	0	1	1
10^3^–10^4^ cells/cm^2^	3	4	2	0	1
10^2^–10^3^ cells/cm^2^	1	1	1	0	0
<10^2^ cells/cm^2^	26	19	30	33	16
Not tested	5	0	5	5	5

a Limit of quantification: 10^2^ cells/cm^2^.

## Discussion

We determined the abundance of mycobacteria and respiratory active mycobacteria in the household water system of healthy volunteers using a culture-independent method. *Mycobacterium* spp. were detected frequently and dominated in bathroom and kitchen drains (Table 2). In these samples, respiratory active *Mycobacterium* spp. were also detected frequently and with relatively high abundance (Table 4). This indicates that drains provide an environmental niche for *Mycobacterium* spp. Drains are present in places such as kitchens and bathrooms to provide egress for water as well as chemicals (like hypochlorite) which controls mildew in damp environments. *Mycobacterium* spp. are relative resistant to acid, alkali [28] and chlorine [29]. The selective pressures of the drain environment, while not suitable for survival of the majority of bacterial species, are no trouble for *mycobacterium* spp. and allow them to dominate. In addition, some protozoa can be reservoir of pathogens. Several bacteria, such as *Legionella pneumophila*, can exploit amoebae as hosts. *Mycobacterium* spp. was also detected in the free-living amoebae isolated from drinking water [30]. *Mycobacterium* spp. is able to survive [31] and proliferate within *Acanthamoeba* spp. [32], [33]. Thus, amoebae may also be a host for *Mycobacterium* spp. *Acanthamoeba* spp. are readily isolated from home sink drains [34], again offering a survival advantage to mycobacterium in this niche environment. If the pathogens are exist in the biofilm formed on the drain, the action of generating aerosol such as cleaning at the drain might lead to expose us to agents.

Feazel *et al*. frequently detected NTM, including *M. avium*, using a culture-independent approach on showerhead biofilms collected from homes and buildings in the United States [4]. Conversely, in this study, we rarely detected *Mycobacterium* spp. from the surface of the showerhead. Nishiuchi *et al*. investigated the genotypes of MAC isolated from patients' and healthy volunteers' residences in Japan [6]. In their report, MAC were not isolated from showerheads in the homes of healthy volunteers and this data is consistent with our results. Many factors affect the ability of *Mycobacterium* spp. to adhere and form a biofilm on various materials [35]. The type of the hot-water supply system used in residences would be different between in Japan and in the United States. The deference of the materials used for showerhead and the type of the hot-water supply system may influence the regional presence of *Mycobacterium* spp. on showerheads.

Interestingly, in this study, we found that *M. avium* was detected only in the bathtub inlet and not in the drain where *Mycobacterium* spp. dominated. We consider that drains may not the source of *M. avium* infection. In general, NTM are thermal resistant bacteria. The thermal resistance of *M. avium* (50–55°C) is higher than that of other NTM such as *M. intracellulare*, *M. kansasii*, *M. marinum*, *M. chelonae* and *M. fortuitum* [36], [37]. A traditional Japanese bathtub has one or two holes which connect to bath-boiler. Water heated by the boiler flows into the bathtub through the hole(s) (bathtub inlet). This system allows the bathtub to fill with hot water and also reheats water in the bathtub. Water temperature in the inlet pipes is 40–60°C. Limited NTM species such as *M. avium,* can survive easily in this environment.

In this study, we determined the abundance of mycobacteria and respiratory active mycobacteria in household water systems. Drains in the bathroom and kitchen sink are the niche for *Mycobacterium* spp. *M. avium* cells are only detected in the bathtub inlet. Our sampling sites were limited (5 sampling sites) and therefore investigation of other places in a household leads to reveal the dynamics of mycobacteria. Yamaguchi *et al*. [38] and Falkinham [37] described the potential impact of human activities on the ecology of nontuberculous mycobacteria. Regional differences also appear to exist as demonstrated by the presence (US) or absence (Japan) of *Mycobacterium* spp. on showerheads. Understanding of the country specific human activities and water usage will help to elucidate the infectious source and route of nontuberculous mycobacterial disease.

## Supporting Information

Figure S1 **Visualization of mycobacterial cells with respiratory activity.** Microscopic image of indigenous bacteria collected from a bathroom drain stained with Auramine O and CTC. Image observed under blue excitation light. Green and red fluorescence are derived from Auramine O and CTC-formazan, respectively. Arrow indicates respiratory active mycobacteria (greenish red).
(PPTX)

Table S1 **Mycobacterial abundance determined with quantitative PCR.**
(XLSX)

Table S2 **Survey for volunteers.**
(XLSX)
