# Supplementary material for: Distribution and Respiratory Activity of Mycobacteria in Household Water System of Healthy Volunteers in Japan
Source: PLoS One. 2014 Oct 28;9(10):e110554. doi: 10.1371/journal.pone.0110554 (PMC4211706; doi:10.1371/journal.pone.0110554)
Supplement: Figure S1 — Visualization of mycobacterial cells with respiratory activity. Microscopic image of indigenous bacteria collected from a bathroom drain stained with Auramine O and CTC. Image observed under blue excitation light. Green and red fluorescence are derived from Auramine O and CTC-formazan, respectively. Arrow indicates respiratory active mycobacteria (greenish red). (PPTX) [file pone.0110554.s001.pptx]

## Slide 1
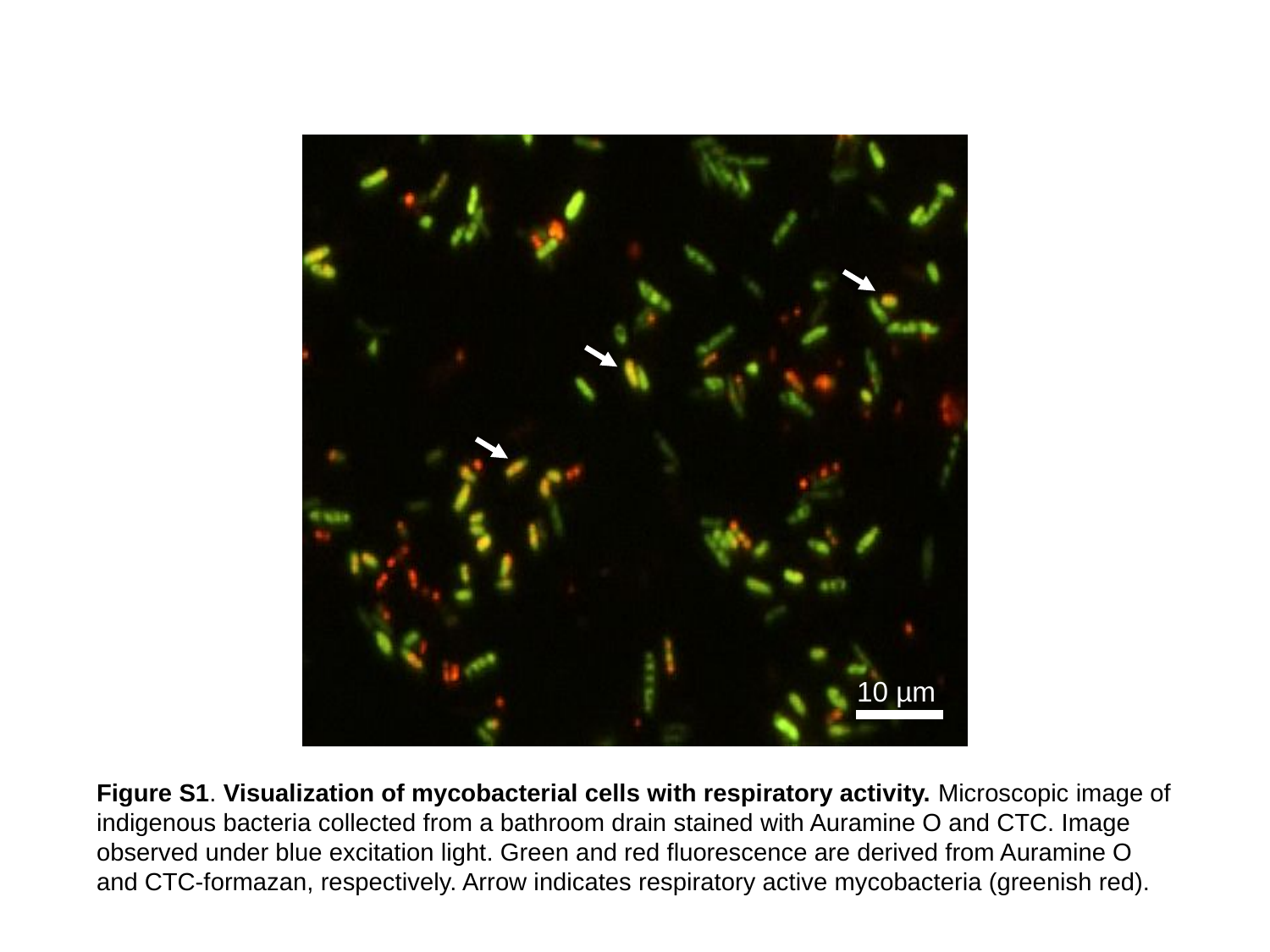

10 µm
Figure S1. Visualization of mycobacterial cells with respiratory activity. Microscopic image of indigenous bacteria collected from a bathroom drain stained with Auramine O and CTC. Image observed under blue excitation light. Green and red fluorescence are derived from Auramine O and CTC-formazan, respectively. Arrow indicates respiratory active mycobacteria (greenish red).
